# Supplementary material for: Spermidine/Spermine N1-Acetyltransferase 1 (SAT1)—A Potential Gene Target for Selective Sensitization of Glioblastoma Cells Using an Ionizable Lipid Nanoparticle to Deliver siRNA
Source: Cancers (Basel). 2022 Oct 22;14(21):5179. doi: 10.3390/cancers14215179 (PMC9656607; doi:10.3390/cancers14215179)
Supplement: Supplementary file 1 [file cancers-14-05179-s001.zip › cancers-1601031-supplementary.pdf]

Spermidine/spermine N1-acetyltransferase 1 (*SAT1*) – A Gene Target for Selective Sensitization of GB Cells using an Ionizable Lipid Nanoparticle to Deliver siRNA.

Vinith Yathindranath, Nura Safa, Babu V. Sajesh, Kelly Schwinghamer, Magimairajan Issai Vanan, Rashid Bux, Daniel S. Sitar, Marshall Pitz, Teruna J. Siahaan, and Donald W. Miller

SUPPORTING INFORMATION

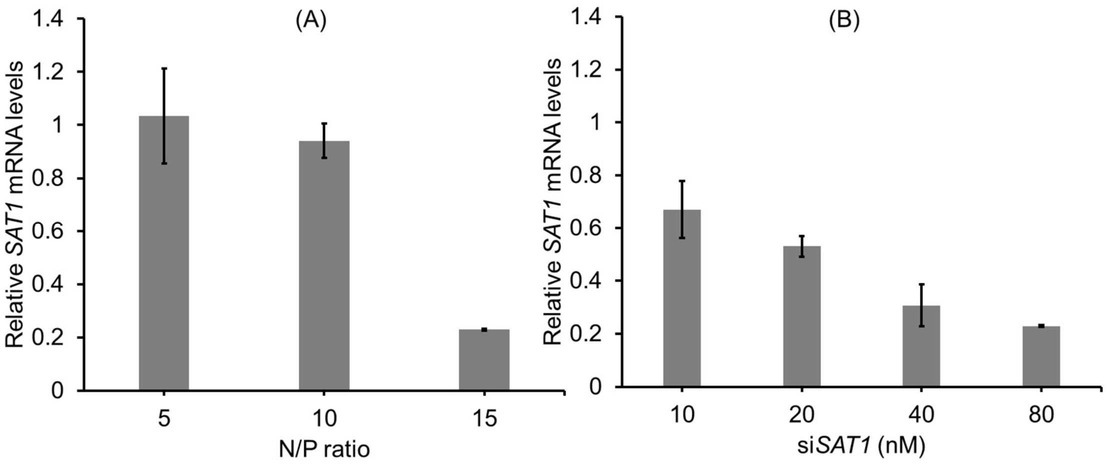

Figure S1. *SAT1* knockdown efficiency of LNP-si*SAT1* (si*SAT1* 80 nM with 1 μg/mL APOE) with different N/P (amine groups in DODAP/phosphate groups in si*SAT1*) ratio (A) and SAT1 knockdown efficiency of LNP-si*SAT1* (N/P = 15) at different concentration (B) in U251 cells compared to control cells that received LNP-siSCR. For all the preparations, the U251 cells cultured in 12 well plate (20,000/cm^2) was transfected in the presence of 1 μg/mL APOE. The *SAT1* mRNA levels were determined 48-hours after transfection (n = 3).

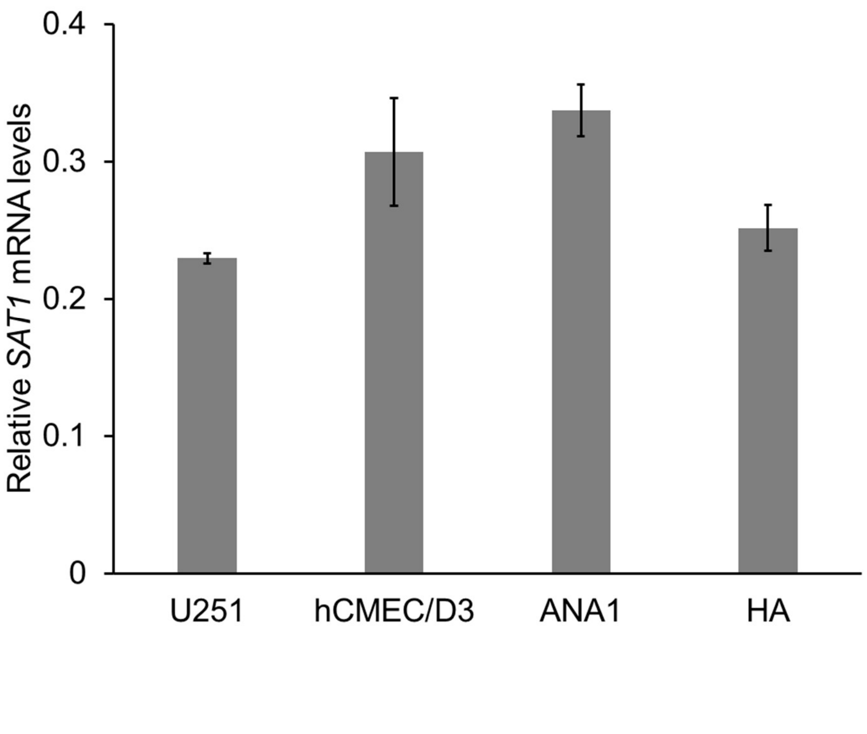

Figure S2. Relative *SAT1* mRNA expression level in LNP-si*SAT1* transfected GBM (U251), brain endothelial (hCMEC/D3), microglia (ANA-1) and astrocyte (HA) cells compared to control cells which received LNP-siSCR. Expression was determined 48-hours after treatment and represent the mean + SEM (n = 3). .

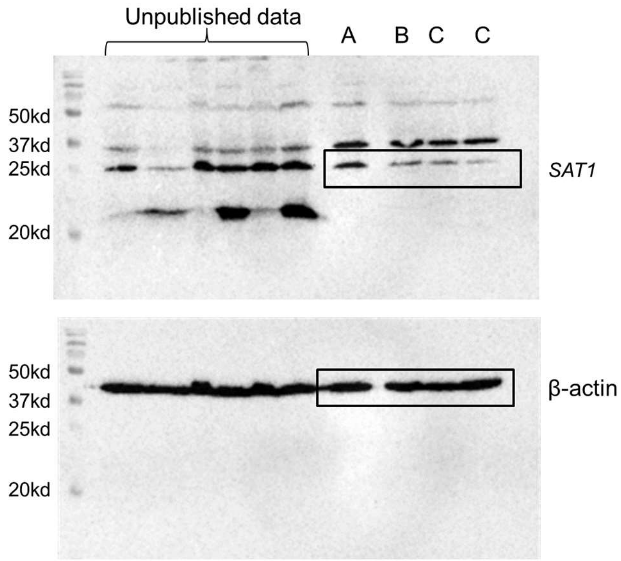

Bands	Densitometry readings			
	A	B	C	C
SAT1	168.26	93.56	90.04	53.50
β-actin	226.30	230.27	218.68	227.69

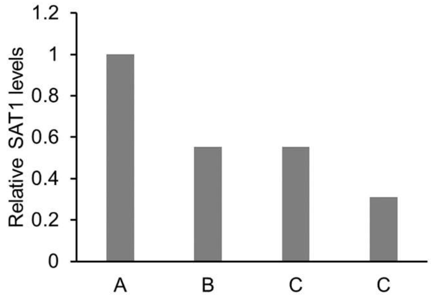

Figure S3. Left panel: The whole blot for the bands (highlighted) shown in Figure 2C of the manuscript. The top and bottom images show the immunoblotting for *SAT1* and β-actin from the same membrane; A – Control, B and C – LNP-si*SAT1* 40 and 80 nM, respectively. Right panel: The densitometry readings and relative *SAT1* levels calculated from the highlighted bands on the blots from the left panel.

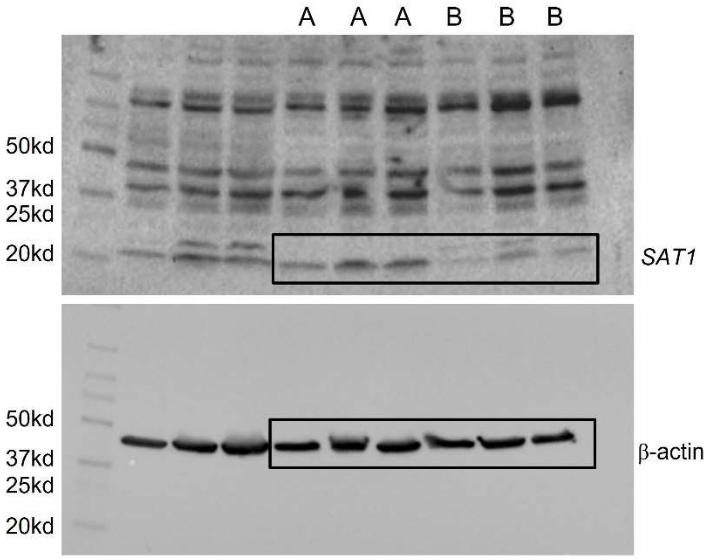

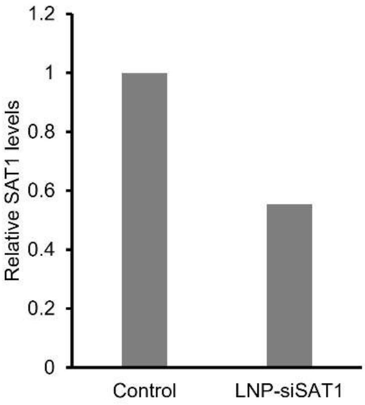

Figure S4. Left panel: The whole blot for the bands (highlighted) shown in Figure 2F of the manuscript. The top and bottom images show the immunoblotting for *SAT1* and β-actin from the same membrane; A – Control and B – LNP-si*SAT1* (80 nM). Right panel: The relative *SAT1* levels calculated from the highlighted bands on the blots from the left panel.

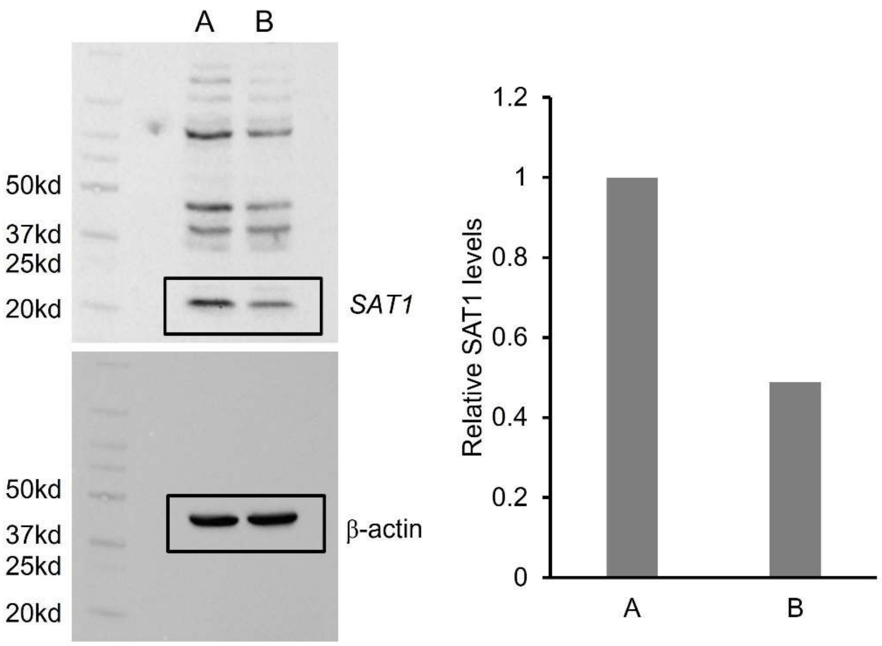

Figure S5. Left panel: The whole blot for the bands (highlighted) shown in Figure 2G of the manuscript. The top and bottom images show the immunoblotting for *SAT1* and β-actin from the same membrane; A – Control and B – LNP-si*SAT1* (80 nM). Right panel: The relative *SAT1* levels calculated from the highlighted bands on the blots from the left panel.

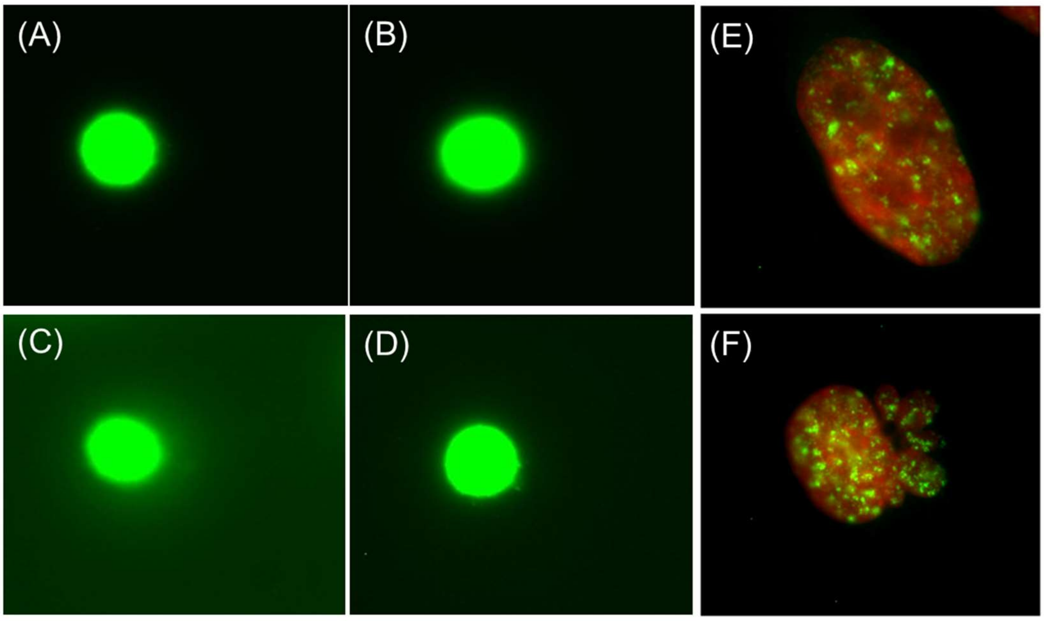

Figure S6. Single cell gel electrophoresis (comet assay) of U251 cells treated with LNP-siSCR (A) and LNP-si*SAT1*(B) that were not exposed to radiation. Single-cell gel electrophoresis (comet assay) on U251 cells treated with LNP-siSCR (C) and LNP-si*SAT1* (D) 24-hours after 10 Gy radiation. The reduction in the observable tail moment as compared to 6-hours could be attributed to the completion of the DNA damage repair. γ-H2AX (green) immunofluorescence and DAPI overlay of LNP-siSCR (E) and LNP-si*SAT1* (F) cells 6-hrs following exposure to 10 Gy radiation. A considerably higher amount of γ-H2AX foci compared to 1 Gy radiation was observed, indicating that the DNA DSB is radiation dose-dependent.
